# Impact of Sleep Quality on Gait Variability: Pilot Cohort Study

**DOI:** 10.2196/81630

**Published:** 2026-06-16

**Authors:** Francesca R Marino, Minzae Kim, Phillip H Hwang, Yorghos Tripodis, Michael Alosco, Jesse Mez, Ann McKee, Rhoda Au, Robert Joseph Thomas, Ludy Shih

**Affiliations:** 1 Department of Anatomy and Neurobiology, Chobanian and Avedisian School of Medicine, Boston University, 72 E Concord Street, Boston, MA, 02118, United States, 1 617-206-6148; 2 Department of Medicine, Chobanian and Avedisian School of Medicine, Boston University, Boston, MA, United States; 3 Department of Epidemiology, School of Public Health, Boston University, Boston, MA, United States; 4 The Framingham Heart Study, Chobanian and Avedisian School of Medicine, Boston University, Boston, MA, United States; 5 Alzheimer's Disease Research Center and CTE Center, Chobanian and Avedisian School of Medicine, Boston University, Boston, MA, United States; 6 Department of Biostatistics, School of Public Health, Boston University, Boston, MA, United States; 7 Department of Neurology, Chobanian and Avedisian School of Medicine, Boston University, Boston, MA, United States; 8 VA Bedford Healthcare System, US Department of Veteran Affairs, Bedford, MA, United States; 9 Department of Pathology and Laboratory Medicine, Chobanian and Avedisian School of Medicine, Boston University, Boston, MA, United States; 10 Department of Medicine, Division of Pulmonary, Critical Care and Sleep Medicine, Beth Israel Deaconess Medical Center, Harvard Medical School, Boston, MA, United States; 11 Department of Neurology, Beth Israel Deaconess Medical Center, Harvard Medical School, Boston, MA, United States

**Keywords:** sleep, gait, variability, wearables, sensors, older adults

## Abstract

**Background:**

Higher step width variability while walking is associated with poor physical function and falls. Sleep is an established modifiable risk factor for both gait and physical function impairments, but it remains to be examined whether sleep is also related to step width variability.

**Objective:**

This study aimed to evaluate the cross-sectional associations between objectively measured sleep quality, using cardiopulmonary coupling spectrograms, and step width variability during a preferred walking condition among middle-aged and older adults.

**Methods:**

This study included 72 adults (mean age 71, SD 8.3 y; n=37, 51% female; n=65, 90% non-Hispanic White) who had ≥2 nights of objectively measured sleep (cardiopulmonary coupling via SleepImage ring) and completed a 10-m walk at preferred speed while wearing inertial sensors (APDM Mobility Lab). Sleep measures included sleep duration, efficiency, fragmentation, stability, apnea-hypopnea index, percentage of time oxygen saturation level <90%, oxygen desaturation index, and respiratory disturbance index. Additional derived sleep variables were explored using least absolute shrinkage and selection operator models. Step width variability was defined by the asymmetry of lateral step variability and categorized as medial (≤−7.5 cm), minimal (within ±7.5 cm; reference), or lateral displacement (≥7.5 cm). Multinomial logistic regression models adjusted for age, sex, race, education, BMI, and usual gait speed evaluated cross-sectional associations between sleep and step width variability categories.

**Results:**

We found that a 1% higher sleep fragmentation was associated with a 6% higher probability of step width variability ≥7.5 cm (95% CI 1.01‐1.11), while a 1% higher sleep stability was associated with a 5% lower probability of variability ≥7.5 cm (95% CI 0.91‐0.99), compared to minimal variability. From the least absolute shrinkage and selection operator models, we found that a 1% higher sleep quality index, a 1% higher rapid eye movement sleep, a 1-second shorter apnea duration, and a 1-beat per minute slower mean heart rate were also associated with a lower probability of lateral compared to minimal displacement.

**Conclusions:**

Poor sleep quality was associated with higher step width variability among middle-aged and older adults. This suggests that sleep may be a modifiable risk factor for maintaining postural stability while walking among middle-aged and older adults. Future studies are needed to examine whether intervening in these sleep measures also lowers the risk of falls.

## Introduction

Ambulating within one’s environment is a complex task that requires cognitive coordination of the motor, vestibular, proprioceptive, and sensory systems [1]. This is typically an automatic task that has low variability [1,2]. Variability in gait reflects stride-to-stride fluctuations in parameters such as gait speed, stride length, step time, and other spatiotemporal characteristics [2]. Gait variability tends to increase with age as ambulation becomes less automatic [3,4]. A key variability measure is step width variability, which reflects dynamic postural control and balance [5-7]. Alterations in gait spatiotemporal characteristics, such as step width variability, impact gait biomechanics. Indeed, variations in step width are associated with alterations in stride time, step length, lower extremity muscle activity, as well as rearfoot, ankle, joint (eg, knee, hip), or trunk kinematics, all of which are related to stability and posture during walking [8]. Step width variability is a marker of poor physical function, as higher variability is associated with slower usual gait speed [9], longer time to complete chair stands [9], and a higher risk of falls [6,10]. Given the high prevalence of falls and physical function impairment among older adults [11,12], studies are needed to identify modifiable risk factors to help maintain step width variability in later life.

Sleep is an established risk factor for both gait and physical function impairments. A recent systematic review found that sleep efficiency, sleep duration, and wake after sleep onset were related to motor outcomes, such as gait speed, time to complete chair stands, and postural control [13]. Among older adults, lower self-reported sleep quality is associated with falls and slower gait speed [14], longer sleep latency is associated with an increased risk of mobility difficulty [15], and shorter sleep duration is associated with declines in gait speed [15]. Self-reported insomnia is also linked with differences in gait speed, stride or step length, and variability in single support, double support, stance, stride or step length, and stride or step time [16]. A study of 49 participants also reported associations between apnea-hypopnea index severity and stride time variability [17]. Most prior studies have focused on gait speed, stride or step length or time variability, or falls, and less is known about the relationships between sleep and step width variability. Many epidemiologic studies also rely on self-reported sleep measures, which may misclassify sleep quality [18,19]. As such, there is a need to evaluate associations between objectively measured sleep quality and step width variability.

Methods to assess sleep have evolved from electroencephalographic (EEG) measurements to actigraphy and autonomic or respiratory signals. One such method is cardiopulmonary coupling (CPC), which integrates electrocortical activity, autonomic activity, and respiration to provide profiles of sleep stability [20,21]. With the increasing availability of wearable devices, the easy assessment of free-living sleep quality is possible. By reducing the need for more traditional in-clinic procedures, such as EEG measurements, wearable devices may also lessen participant burden and improve accessibility [22].

This study aimed to establish associations between sleep and step width variability by leveraging digital data from a clinical sample of middle-aged and older adults. We hypothesized that lower objectively measured sleep quality would be cross-sectionally associated with higher step width variability during a preferred walking speed condition. Given that associations between sleep and gait have been found to differ by sex or cognitive function [14,23,24], we also expected that associations between sleep quality and step width variability would be stronger among men or individuals living with cognitive impairment compared to their respective counterparts.

## Methods

### Participants

This study included participants enrolled in the Boston University (BU) Alzheimer’s Disease Research Center (ADRC) Clinical Core who opted into a digital brain health platform study (Digital Study). In brief, the BU ADRC is one of over 30 ADRCs nationwide that aim to promote collaborative research on Alzheimer’s disease and related dementias. The BU ADRC enrolled adults aged ≥50 years with and without memory complaints who were English-speaking, community-dwelling, and local to the greater Boston area. The BU ADRC sample is representative of a clinic-based sample, including people with concerns about their cognition either because they have cognitive complaints or because they have certain risk factors such as head trauma or a family history. Full inclusion and exclusion criteria are published elsewhere, but participants were generally eligible unless they had a history of major psychiatric or neurologic illness (eg, schizophrenia, bipolar disorder, epilepsy) or head injury with significant loss of consciousness [25].

Starting in 2021, BU ADRC participants have had the option to use various technologies both at home and in the clinic as part of the Digital Study. Participants are given the choice of which technologies they would like to use [22]. This study included Digital Study participants who chose to use both sleep and gait digital technologies. All participants were eligible to complete the sleep and gait assessments. Participants were excluded from the analysis if sleep and gait data were measured >90 days apart (n=2) or if covariate data were missing (n=3). The flowchart for inclusion in the study sample is shown in the *Results* section.

### Ethical Considerations

All study procedures were approved by the Boston University Medical Campus Institutional Review Board (approval number: H-40542). All participants provided written informed consent. The original informed consent and institutional review board approval allowed secondary analysis of the data without requiring additional participant consent. All data analyzed in this study were deidentified. No financial compensation was provided to participants.

### Sleep Measures

#### Recording Process and Automated Analysis

Participants were asked to wear the SleepImage ring (MyCardio LLC) in the free-living environment for at least 3 nights. The ring contains a photoplethysmography sensor that collects heart rate, oxygen saturation (SpO_2_), and actigraphy data. Data were automatically uploaded and processed by SleepImage using the CPC algorithm.

#### Cardiopulmonary Coupling

The CPC method analyzes changes in signals modulated by the autonomic nervous system (eg, heart or pulse rate variability and breathing) to derive sleep stages, states, and a Sleep Quality Index (SQI). The method is based on calculating coupling and coherence between heart or pulse rate variability (eg, time between peaks of consecutive R-waves [RR interval time series]) and fluctuations in R-wave or pulse-wave amplitude induced by respiration to detect changes in breathing (eg, tidal volume variability [TVV]) [20]. These outputs are strongly modulated by sleep-wake state and stage. The cross-spectral power and coherence of the RR time series and corresponding TVV time series are calculated for consecutive windows, and a product of coherence and cross-spectral power is used to obtain the ratio of coherent cross power in the low frequency (0.01‐0.1 Hz) to that in the high-frequency band (0.1‐0.4 Hz). The logarithm of the high-to-low-frequency CPC ratio is then computed to yield a continuous and moving average of overlapping CPC windows and output of stable and unstable nonrapid eye movement (NREM) sleep, REM sleep, and wake. Graphing CPC at relevant frequencies versus time provides the SleepImage spectrogram [20,26]. Stable NREM sleep is characterized by stable breathing and stable oxygenation, high vagal tone, non–cyclic alternating pattern on the EEG, continuous occurrence of slow oscillations, high delta power, blood pressure dipping, and stable arousal threshold. Unstable NREM sleep is a marker of sleep instability and has exactly the opposite features of stable NREM sleep, with variability in TVV, cyclic variation in heart rate, cyclic alternating pattern on EEG, low relative delta power, nondipping of blood pressure, and unstable arousal thresholds. The SQI is a proprietary summary index of the CPC biomarkers of sleep quality, sleep stability, fragmentation, and periodicity, which provides a meaningful unit of measure for sleep health. The SQI is displayed on a scale of 0 to 100 with expected values for both children and adults [27-30]. Sleep spectrograms for participants with higher versus lower SQI values are shown in Figure S1 in Multimedia Appendix 1.

#### Sleep Measures

The following SleepImage measures were identified a priori: (1) sleep duration (hours), defined as the total hours of sleep per night; (2) sleep efficiency (%), defined as the proportion of time asleep out of the total time in bed; (3) sleep fragmentation (%), defined as the probability of transitioning from sleep to wakefulness; (4) stable sleep (%), defined as the proportion of sleep classified as stable (eg, regular breathing, heart rate, etc); (5) apnea-hypopnea index (events/hour), defined as the number of apnea or hypopnea events with ≥4% desaturation per hour; (6) duration (minutes) or percentage (%) of time SpO_2_ levels were <90%; (7) oxygen desaturation index (events/hour), defined as the number of events lasting ≥10 seconds with ≥4% desaturation per hour; and (8) respiratory disturbance index (events/hour), defined as the number of times per hour that breathing was disrupted during sleep. The full methods for calculating these sleep measures are described elsewhere [31]. The remaining variables from the SleepImage report were examined using least absolute shrinkage and selection operator (LASSO) models, as described in the *Statistical Analysis* section.

### Step Width Variability

As part of the in-clinic visits for the Digital Study, participants completed several walking trials while wearing inertial sensors (Opal; APDM) over the L4-5 vertebra, on each wrist, and on each foot. After the sensors were attached, participants walked back and forth down a 10-m hallway at their preferred comfortable (eg, usual) speed for 1 minute. The number of straight walking bouts varied depending on the participant’s usual walking speed. Participants completed this 1-minute walking trial at their usual pace, as well as additional trials at a fast pace, slow pace, and fast-paced dual-task (eg, serial subtractions). Only the usual pace walking trial was included in this analysis. Then, the data were uploaded and processed using Mobility Lab version 2 [32]. Derived variables from these sensors have been validated among healthy adults (mean age 24, SD 6.2 y) [33] and populations with Parkinson disease [34]. These sensors have been used to monitor gait and balance [35-40], and several studies have also evaluated step width variability [36-40].

**Figure 1.**
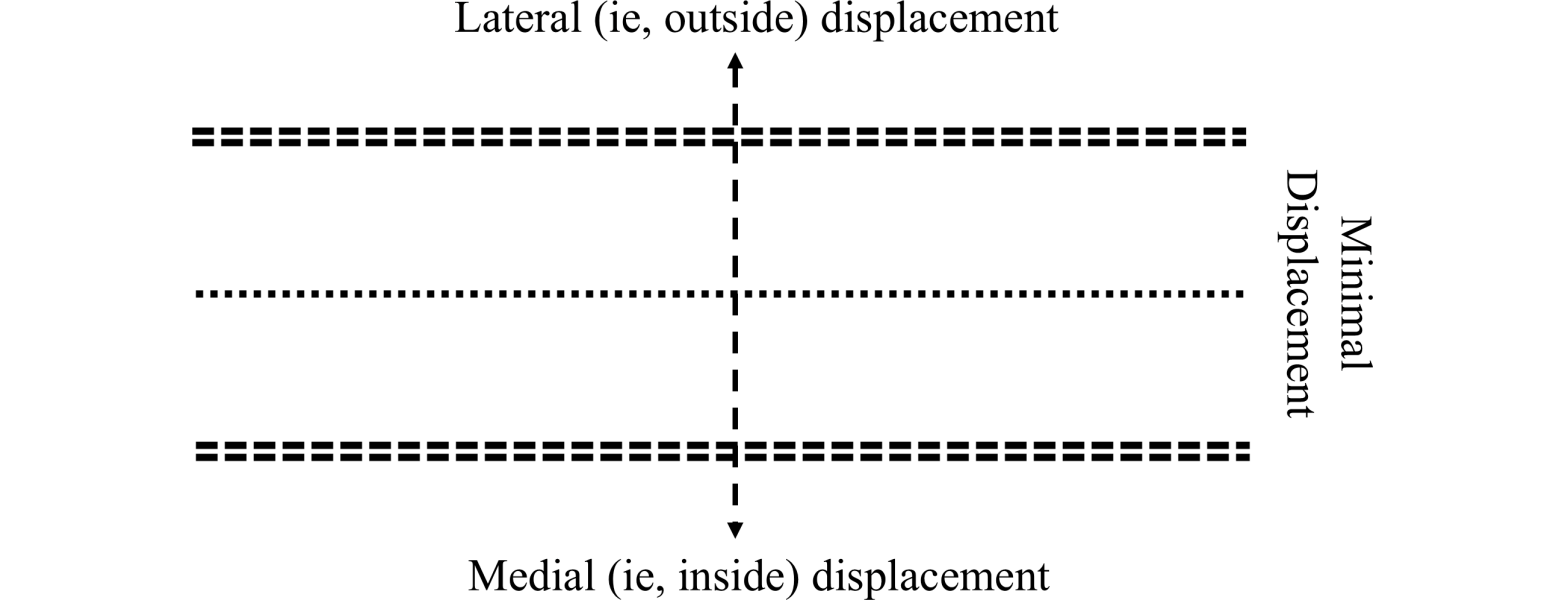
Measurement of asymmetry in lateral step variability by Mobility Lab. For an individual walking along the center dashed line, displacement of the foot to the outside is defined as lateral displacement, displacement of the foot to the inside is defined as minimal displacement, and displacement of the foot within the outside and inside limits is defined as minimal displacement.

The outcome of interest was step width variability, as measured by asymmetry in lateral step variability during the preferred comfortable speed walking trial. This was defined as the variability of perpendicular deviations of the middle foot placement from the line connecting the first and third placements, when considering 3 consecutive foot placements made by the same foot [32]. Negative values indicated movement to the inside (eg, medial displacement), whereas positive values indicated movement to the outside (eg, lateral displacement; Figure 1) [32]. Mobility Lab 2 generated 1 summary value of asymmetry in lateral step variability. Mobility Lab 2 generated separate derived variables for information related to turning during the walking trials, which were not used in this analysis. For this study, variability was categorized as displacement within ±7.5 cm (eg, minimal displacement), ≤−7.5 cm (eg, medial displacement), or ≥7.5 cm (eg, lateral displacement). To determine whether greater displacement to either the inside or outside was associated with poor sleep quality, we also calculated the absolute value of asymmetry lateral step variability and analyzed this continuously (eg, higher values reflecting greater displacement in either direction).

### Covariates

Covariates were measured at each ADRC visit as part of the National Alzheimer’s Coordinating Center Uniform Data Set [41]. Age was measured in years. Sex was categorized as male or female. Race was categorized as White, Black, American Indian or Alaska Native, Native Hawaiian or Other Pacific Islander, Asian, other, or unknown. Participants in the current sample identified as White, Black, or Asian. Due to small sample sizes, participants were classified as White or either Black or Asian. Educational attainment (years) was self-reported. BMI was measured as body mass (kg) divided by squared height (m^2^). Usual gait speed (m/s) was measured by the APDM sensors [32]. Cognitive status was measured by the Clinical Dementia Rating (CDR) [42]. Participants were classified as having scores of 0 (n=56) or ≥0.5 (n=16; range 0.5‐2.5).

### Statistical Analysis

Sample characteristics were compared overall and by categories of step width variability. Means and SDs or counts and percentages were calculated for continuous or categorical variables, respectively.

Cross-sectional associations between each a priori sleep measure and categories of step width variability were estimated using separate multinomial logistic regression models. These models estimated the relative risk ratio (RRR) and 95% CIs for medial or lateral displacement compared to minimal displacement (eg, reference group). RRR is a measure of association that estimates the probability of being in a particular outcome category (eg, medial or lateral displacement) relative to the reference category (eg, minimal displacement), per 1-unit increase in the exposure variable (eg, sleep measure). All models were adjusted for age, sex, race, education, BMI, and usual gait speed.

LASSO models evaluated the remaining SleepImage features to identify additional sleep variables for differentiating step width variability categories. In brief, this method is a machine learning approach that selects a subset of independent variables (eg, sleep) that best describe a dependent variable (eg, step width variability). A regularization process is applied to shrink the coefficients of variables that are less related to the outcome to zero. Then, variables with a nonzero coefficient are selected. This method is particularly useful for providing higher prediction accuracy and reducing the risk of overfitting in datasets with smaller sample sizes and a larger number of possible variables [43]. The features identified from the LASSO model were examined in separate multinomial regression models adjusted for the same covariates.

We also conducted several sensitivity analyses. First, to determine whether associations differed by certain individual-level characteristics, we stratified each model by sex (men vs women) or cognitive status (CDR of 0 versus ≥0.5). Second, to examine whether poor sleep was associated with higher step width variability irrespective of direction (eg, medial or lateral), we estimated the cross-sectional associations between sleep measures and the absolute value of step width variability using linear regression models. Finally, to evaluate whether associations were driven by extreme values, we repeated the primary analyses after excluding 1 participant with step width variability >57 cm.

The significance level for the multinomial logistic regression models was set to *P*<.05. Due to the number of comparisons, we applied a Benjamini-Hochberg false discovery rate correction (false discovery rate–corrected *P* value [pFDR]) for models estimating associations between either the a priori (9 comparisons) or LASSO-identified (5 comparisons) sleep features with step width variability categories [44]. All analyses were performed using Stata (version 18.0; StataCorp). Figures were generated using R (version 4.2.2).

## Results

### Sample Characteristics

**Figure 2.**
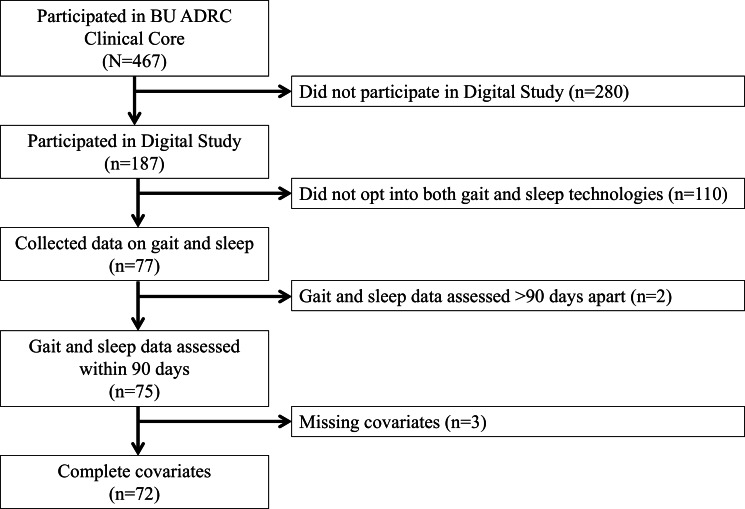
Flowchart for inclusion in the analytic sample. BU ADRC: Boston University Alzheimer’s Disease Research Center.

Of the 72 participants in this sample (Figure 2), the mean age was 71 (SD 8.3; range 52‐88) years, 51% (n=37) were female, 90% (n=65) were non-Hispanic White, and 22% (n=16) had CDR scores ≥0.5. On average, asymmetry in lateral step variability was 1.92 (SD 20.54) cm, with a range of −38.85 to 57.91 cm. For step width variability categories, 31% (n=22) had minimal, 29% (n=21) had medial, and 40% (n=29) had lateral displacement. Participants with minimal displacement (eg, step width variability within ±7.5 cm) were older; more likely to be White; had higher educational attainment; slower usual gait speed; and lower prevalence of hypertension, diabetes, or cognitive impairment compared to those with medial (eg, ≤−7.5 cm) or lateral (eg, ≥7.5 cm) displacement. However, none of the differences in sample characteristics across step width variability groups were statistically significant (Table 1).

**Table 1.** Sample characteristics overall and by step width variability categories.

Characteristics	Overall (N=72)	Minimal^a^ (n=22)	Medial^a^ (n=21)	Lateral^a^ (n=29)	*P* value
Demographics					
Age (y), mean (SD)	71.0 (8.3)	72.2 (9.9)	70.8 (7.4)	70.2 (7.7)	.71
Female, n (%)	37 (51)	11 (50)	9 (43)	17 (59)	.54
Non-Hispanic White, n (%)	65 (90)	22 (100)	19 (90)	24 (83)	.12
Education (y), mean (SD)	16.7 (2.1)	17.5 (1.8)	16.2 (1.8)	16.4 (2.5)	.09
Married, n (%)	53 (74)	16 (73)	16 (76)	21 (72)	.63
Medical conditions, n (%)					
Hypertension	24 (33)	6 (27)	9 (43)	9 (31)	.52
Diabetes	5 (7)	0 (0)	2 (10)	3 (10)	.30
Sleep apnea	22 (31)	7 (32)	7 (33)	8 (28)	.90
Sleep disturbances	10 (14)	4 (18)	3 (14)	3 (10)	.72
CDR^b^ ≥0.5	16 (22)	3 (14)	6 (29)	7 (24)	.47
Gait speed (m/s), mean (SD)	1.04 (0.17)	1.01 (0.17)	1.08 (0.14)	1.04 (0.19)	.46
Timing, mean (SD)					
Nights of sleep (n)^c^	6.4 (6.0)	7.2 (7.3)	6.5 (5.9)	5.8 (5.0)	.70
Time lag (days)^d^	12.9 (16.1)	14.0 (19.1)	12.0 (10.7)	12.6 (17.5)	.92

a Minimal displacement within ±7.5 cm; medial displacement ≤−7.5 cm; and lateral displacement ≥7.5 cm during the preferred walking speed condition.

b CDR: Clinical Dementia Rating scale.

c Number of nights of sleep data.

d Number of days between sleep and step width variability measurements.

### Cross-Sectional Associations Between Sleep and Step Width Variability Categories

Of the sleep features identified a priori, sleep fragmentation and stable sleep percentage were associated with the probability of having high step width variability. After adjustment for age, sex, race, education, BMI, and usual gait speed, a 1% higher sleep fragmentation was associated with a 6% higher probability of lateral displacement, and a 1% higher stable sleep percentage was associated with a 5% lower probability of lateral displacement compared to minimal displacement (fragmentation: RRR 1.06, 95% CI 1.01‐1.11; stable sleep: RRR 0.95, 95% CI 0.91‐0.99; Table 2). However, these findings were not statistically significant after FDR correction (Table S1 in Multimedia Appendix 1). There were no associations between sleep duration, sleep efficiency, apnea-hypopnea index, SpO_2_ <90%, oxygen desaturation index, or respiratory disturbance index with step width variability categories or between any sleep measure and probability of medial displacement (Table 2). The results were unchanged after excluding 1 participant with step width variability >57 cm (data not shown).

**Table 2.** Cross-sectional associations between a priori sleep and step width variability categories (N=72).

Sleep variable	Medial^a^^,^^b^ (RRR^c^, 95% CI)	Lateral^a^^,^^b^ (RRR, 95% CI)
Sleep duration (h)	1.07 (0.73-1.59)	0.86 (0.59-1.26)
Sleep efficiency (%)	1.02 (0.96-1.09)	0.99 (0.94-1.05)
Sleep fragmentation (%)	1.00 (0.96-1.06)	1.06 (1.01-1.11)
Stable sleep (%)	1.01 (0.96-1.06)	0.95 (0.91-0.99)
AHI^d^ (numbers per hour)	1.01 (0.94-1.08)	1.00 (0.94-1.08)
SpO_2_^e^ <90% (minute)	1.00 (0.98-1.01)	0.99 (0.98-1.01)
SpO_2_ <90% (%)	0.99 (0.93-1.05)	0.96 (0.90-1.03)
ODI^f^ (numbers per hour)	1.02 (0.95-1.09)	1.01 (0.94-1.08)
RDI^g^ (numbers per hour)	1.00 (0.95-1.06)	1.01 (0.96-1.07)

a Minimal displacement within ±7.5 cm (reference); medial displacement ≤−7.5 cm; and lateral displacement ≥7.5 cm during the preferred walking speed condition.

b Model adjusted for age, sex, race, education, BMI, and usual gait speed.

c RRR: relative risk ratio.

d AHI: apnea-hypopnea index.

e SpO_2_: oxygen saturation.

f ODI: oxygen desaturation index.

g RDI: respiratory disturbance index.

### Sleep Measures Identified by LASSO Models

The LASSO model identified SQI, REM sleep percentage, mean SpO_2_, minimum apnea duration, and mean heart rate as being related to lateral compared to minimal displacement. After full covariate adjustment, 1% higher SQI and 1% higher REM sleep were associated with lower probability of lateral displacement, whereas a 1-second longer minimum apnea duration and a 1 beat per minute faster mean heart rate were associated with higher probability of lateral displacement compared to minimal displacement (SQI: RRR 0.92, 95% CI 0.87‐0.98; REM sleep: RRR 0.87, 95% CI 0.77‐0.99; apnea duration: RRR 2.34, 95% CI 1.06‐5.16; heart rate: RRR 1.14, 95% CI 1.03‐1.26; Table 3). The findings related to SQI were robust at the trend-level after FDR correction (*P*=.09; Table S2 in Multimedia Appendix 1). The LASSO model did not identify any sleep measures associated with medial displacement. However, 1 beat per minute faster mean heart rate was associated with 13% higher probability of medial compared to minimal displacement (95% CI 1.01‐1.25; Table 3). The results were unchanged after excluding 1 participant with step width variability >57 cm (data not shown).

**Table 3.** Cross-sectional associations between the additional sleep measures identified from least absolute shrinkage and selection operator (LASSO) and step width variability categories (N=72).

Sleep variable	Medial^a^^,^^b^ (RRR^c^, 95% CI)	Lateral^a^^,^^b^ (RRR, 95% CI)
SQI^d^ (%)	1.00 (0.95-1.05)	0.92 (0.87-0.98)
REM^e^ sleep (%)	0.91 (0.81-1.03)	0.87 (0.77-0.99)
Mean SpO_2_^f^ (%)	1.01 (0.69-1.48)	1.31 (0.90-1.92)
Minimum apnea duration (s)	1.35 (0.59-3.05)	2.34 (1.06-5.16)
Mean heart rate (BPM^g^)	1.13 (1.01-1.25)	1.14 (1.03-1.26)

a Minimal displacement within ±7.5 cm (reference); medial displacement ≤−7.5 cm; and lateral displacement ≥7.5 cm during the preferred walking speed condition.

b Model adjusted for age, sex, race, education, body mass index, and usual gait speed.

c RRR: relative risk ratio.

d SQI: sleep quality index.

e REM: rapid eye movement.

f SpO_2_: oxygen saturation.

g BPM: beats per minute.

### Effect Measure Modification by Sex

Among men (n=35), the findings were robust for stable sleep percentage, SQI, and REM sleep percentage. All other primary findings were attenuated, but the magnitude of the effect sizes was consistent with the primary analyses. Among women (n=37), the findings were robust for mean heart rate. All other primary findings were attenuated, but the magnitude of the effect sizes was consistent with the primary analyses (Table S3 in Multimedia Appendix 1).

### Effect Measure Modification by Cognitive Status

The models among participants with CDR ≥0.5 did not converge due to small sample sizes with the addition of usual gait speed as a covariate. Therefore, these models were adjusted for age, sex, race, education, and BMI. Among participants with normal cognition (CDR=0; n=56), the findings were robust for SQI. All other findings were attenuated, although the magnitude of the effect sizes was consistent with the primary analyses. Among participants with cognitive impairment (CDR ≥0.5; n=16), all findings were attenuated. The magnitude of the effect size was similar for sleep fragmentation and mean heart rate (Table S4 in Multimedia Appendix 1).

### Cross-Sectional Associations Between Sleep and the Absolute Value of Step Width Variability

After taking the absolute value of step width variability, the mean was 16.63 (SD 12.05) cm, with a range of 0.19 to 57.91 cm. After covariate adjustment, 1% longer REM sleep was associated with 0.81 cm lower step width variability (95% CI −1.32 to −0.30). There were no associations with any other sleep measures (Table S5 in Multimedia Appendix 1).

## Discussion

### Principal Results

In this clinical sample of middle-aged and older adults, lower objectively measured sleep quality was cross-sectionally associated with higher step width variability during the preferred speed walking condition. Specifically, lower SQI, shorter stable or REM sleep, higher sleep fragmentation, longer apnea duration, and faster mean heart rate were each associated with a higher probability of having lateral (eg, ≥7.5 cm) versus minimal displacement (eg, within ±7.5 cm). The associations between sleep and step width variability were also independent of usual walking pace. Overall, these findings suggest that sleep may be a modifiable risk factor for minimizing step width variability. This has potentially important implications for preventing falls and physical function decline in later life.

This study found that several objectively measured sleep variables related to lower sleep quality were associated with higher step width variability. Most prior studies have focused on associations between sleep and gait speed [14,15,23,24], despite evidence that measures of gait variability may be stronger risk markers for falls [45]. Postural control, which is related to step width variability [2], is impaired among individuals who experience sleep deprivation [46] or sleep-disordered breathing [47]. Additionally, studies have evaluated other measures of gait variability, such as variability in stride length, step length, stride time, or step time. Indeed, lower sleep efficiency, the presence of insomnia, or sleep-related breathing disorders are associated with higher variability in these measures [13,16,17]. This study builds on existing evidence to suggest that sleep quality, even without a sleep disorder, may be an important modifiable risk factor for gait dysfunction, including higher step width variability.

There are several possible mechanisms through which sleep may impact gait and step width variability. Gait is a complex task that requires coordination of several systems [48,49]. Brain regions, including the prefrontal cortex, basal ganglia, brainstem, and cerebellum, are responsible for receiving information about body positioning from the visual, vestibular, and proprioceptive systems. These brain regions must interpret afferent signals and coordinate a motor response through the pyramidal tract, peripheral nerves, and neuromuscular system [48,49]. Further, step width variability is a measure of postural control during locomotion [2], and dynamic postural control is likely mediated through spinal pathways, the cerebellum, the sensorimotor thalamus, the supplementary motor area, and the premotor cortex [50]. Evidence links intermittent hypoxia, which occurs during sleep apnea, with damage to the prefrontal cortex and cerebellum [51], as well as sleep apnea with incident cognitive impairment [52]. Poor sleep quality is also associated with the dysregulation of synaptic homeostasis and the glymphatic system, a higher burden of cerebrovascular disease (eg, white matter hyperintensities) and amyloid-β, and worse cognitive performance [53]. While the role of the basal ganglia in normal sleep mechanisms is less well defined than cortical and brainstem neural circuits, the profound changes associated with disorders of the basal ganglia suggest key roles [54]. These findings may reflect alterations of neural networks from cortical to spinal cord levels, while cortex-to-brainstem areas are more involved in sleep regulation, including the interactions mapped by CPC outputs.

These mechanisms align with prior studies linking poor sleep with gait dysfunction. First, sleep deprivation is associated with poorer postural control among older adults [55]. Sleep deprivation among college students is also related to lower metabolic activity in the prefrontal cortex, basal ganglia, cerebellum, and thalamus, as well as greater difficulty synchronizing gait to an auditory signal [56]. Second, sleep disruption is associated with reduced gait complexity (eg, less adaptability to the environment) [57], indicating greater regularity of stride-to-stride fluctuations [58]. Finally, sleepiness due to poor sleep quality may be related to gait dysfunction. This is supported by several studies linking cumulative exposure to drugs with sedative properties to decreased gait regularity, pace, and complexity [45,59], as well as self-reported sleepiness with a higher risk of falls [51]. Additional research is needed to identify the specific mechanisms through which sleep may impact step width variability.

In this study, associations between sleep quality and step width variability were stronger among men than women. Indeed, several findings were robust for male participants, while only associations with mean heart rate persisted among female participants. However, the magnitude of the effect sizes was similar for both men and women. This aligns with a prior study that found that slower gait speed was cross-sectionally associated with poor sleep quality and longer sleep duration (eg, >8 h) among men but not women [23]. One potential mechanism explaining the links between sleep and gait among men is that poor sleep quality leads to reductions in muscle mass and strength. This mechanism is supported by a study finding that grip strength mediates associations between self-reported sleep quality and both gait speed and falls among men but not women [14]. Though replication is needed, these preliminary findings suggest that men may be particularly susceptible to higher step width variability related to poor sleep.

Contrary to our hypothesis, the findings for SQI were robust among participants with normal cognition, while no findings were statistically significant among those with cognitive impairment. Given that cognitive decline and dementia are associated with slower gait speed and abnormal gait patterns [4,60], we hypothesized that individuals with cognitive impairment would be more susceptible to gait dysfunction related to poor sleep quality. Populations living with cognitive impairment, such as Alzheimer or Parkinson disease, have higher gait variability [61], lower postural control [61], and a higher risk of falls [62] than cognitively intact controls. As such, we would expect that lower step width variability may be of particular importance for postural control and balance during walking among those with cognitive impairment. Additionally, slower gait speed is linked with lower global cognition among individuals with sleep apnea [24] and lower executive function or attention [49]. The combination of slow gait and poor sleep duration is also associated with higher odds of cognitive impairment [63]. There are a few possible explanations for why we did not observe associations between sleep and step width variability among participants with cognitive impairment in our study. First, we used a global measure of cognitive impairment, but specific cognitive domains, particularly attention and executive function, are linked to both gait and sleep [49,64,65]. Sleep may be more strongly related to step width variability among those with impairment in these domains. Second, the system coordinating gait may have already been disrupted among those with cognitive impairment, potentially attenuating the added harm from poor sleep quality. Finally, we may have been underpowered to detect associations with global cognitive impairment or within specific cognitive domains due to the modest sample size. The replication of the findings is needed in larger, more diverse cohorts.

This study supports the feasibility of remotely monitoring risk factors using digital technologies in the free-living environment. Integrating digital technologies into research has several advantages, including improved feasibility and accessibility, as well as reduced participant burden [22]. Data from these technologies provide additional information about an individual’s free-living behaviors and function. Indeed, studies have criticized self-reported questionnaires for misclassifying sleep quality [18,19]. The digital acquisition of gait characteristics in this study also allowed for more nuanced measures of gait characteristics, including step width variability. While this study is limited by a modest sample size and cross-sectional design, these findings support continued research to identify digital sleep biomarkers that relate to functional outcomes. Future studies are also needed to evaluate whether these digital sleep measures also relate to the risk of falls or physical function decline (eg, grip strength, lower extremity strength, balance) and to test whether intervening in these sleep characteristics improves gait and physical function.

### Limitations and Strengths

This study has several important limitations. First, there is a lack of consensus on cut points to define minimal, medial, and lateral displacement. Future studies are needed to validate these cut points for asymmetry in lateral step variability. Second, there was a gap in time between measurement of sleep and step width variability. We attempted to address this by excluding participants with measurements >90 days apart. Third, the BU ADRC is a clinical cohort that may be less representative compared to population- or community-based samples. This sample also had limited racial and ethnic diversity, including variations in skin pigmentation, which can bias optoelectronic sensing (eg, plethysmography sensors). Future studies with larger, more diverse samples are needed to evaluate generalizability and sensor performance. Fourth, the sample size was modest, and some analyses may be underpowered. This study is a first step toward establishing associations between sleep and step width variability. Finally, there is a possibility of residual or unmeasured confounding. A major strength of this study is the use of objectively measured sleep quality and step width variability. The use of digital tools provided nuanced measures of both our exposure and outcome. Machine learning methods were also leveraged for sleep feature selection, reducing the risk of overfitting given the modest sample size [43]. Additionally, data were available on cognitive function and important covariates.

### Conclusions

This study found that poor objectively measured sleep quality was associated with higher step width variability during preferred speed walking. Future studies are needed to evaluate whether monitoring, or possibly intervening in, these sleep measures could help to minimize the risk of falls or physical function decline in later life.

## Supplementary material

10.2196/81630 Multimedia Appendix 1 Results of secondary and sensitivity analyses.

## Abbreviations

ADRC: Alzheimer’s Disease Research Center
BU: Boston University
CDR: Clinical Dementia Rating
CPC: cardiopulmonary coupling
EEG: electroencephalogram
FDR: false discovery rate
LASSO: least absolute shrinkage and selection operator
NREM: nonrapid eye movement
RRR: relative risk ratio
SpO_2_: oxygen saturation
SQI: sleep quality index
TVV: tidal volume variability
