# Supplementary material for: Impact of Sleep Quality on Gait Variability: Pilot Cohort Study
Source: JMIR Aging. 2026 Jun 16;9:e81630. doi: 10.2196/81630 (PMC13271590; doi:10.2196/81630)
Supplement: Multimedia Appendix 1 [file aging-v9-e81630-s001.docx]

**Supplementary Files**

Supplemental Table 1. Cross-Sectional Associations between *A Priori* Sleep and Step Width Variability Categories, Corrected for False Discovery Rate (N=72)

|  | **Minimal ^a,b^** | **Medial ^a,b^** | | **Lateral ^a,b^** | |
| --- | --- | --- | --- | --- | --- |
|  | **(RRR, 95% CI)** | **(RRR, 95% CI)** | **pFDR** | **(RRR, 95% CI)** | **pFDR** |
| Sleep Duration (hr) | REF | 1.07 (0.73, 1.59) | 0.99 | 0.86 (0.59, 1.26) | 0.99 |
| Sleep Efficiency (%) | REF | 1.02 (0.96, 1.09) | 0.99 | 0.99 (0.94, 1.05) | 0.99 |
| Sleep Fragmentation (%) | REF | 1.00 (0.96, 1.06) | 0.99 | 1.06 (1.01, 1.11) | 0.28 |
| Stable Sleep (%) | REF | 1.01 (0.96, 1.06) | 0.99 | 0.95 (0.91, 0.99) | 0.50 |
| AHI (#/hr) | REF | 1.01 (0.94, 1.08) | 0.99 | 1.00 (0.94, 1.08) | 0.99 |
| SpO2 < 90% (min) | REF | 1.00 (0.98, 1.01) | 0.99 | 0.99 (0.98, 1.01) | 0.99 |
| SpO2 < 90% (%) | REF | 0.99 (0.93, 1.05) | 0.99 | 0.96 (0.90, 1.03) | 0.99 |
| ODI (#/hr) | REF | 1.02 (0.95, 1.09) | 0.99 | 1.01 (0.94, 1.08) | 0.99 |
| RDI (#/hr) | REF | 1.00 (0.95, 1.06) | 0.99 | 1.01 (0.95, 1.06) | 0.99 |

Note: pFDR=false discovery rate-corrected p-value; AHI=apnea hypopnea index; ODI=oxygen desaturation index; RDI=respiratory disturbance index

a. Minimal displacement within ± 7.5 cm; medial displacement ≤ -7.5 cm; lateral displacement ≥ 7.5 cm during preferred walking speed condition

b. Model adjusted for age, sex, race, education, body mass index, and usual gait speed

Supplemental Table 2. Cross-Sectional Associations between the Additional Sleep Measures Identified from LASSO and Step Width Variability Categories, Corrected for False Discovery Rate (N=72)

|  | **Minimal ^a,b^** | **Medial ^a,b^** | | **Lateral ^a,b^** | |
| --- | --- | --- | --- | --- | --- |
|  | **(RRR, 95% CI)** | **(RRR, 95% CI)** | **pFDR** | **(RRR, 95% CI)** | **pFDR** |
| SQI (%) | REF | 1.00 (0.95, 1.05) | 0.99 | 0.92 (0.87, 0.98) | 0.09 |
| REM Sleep (%) | REF | 0.91 (0.81, 1.03) | 0.99 | 0.87 (0.77, 0.99) | 0.45 |
| Mean SpO_2_ (%) | REF | 1.01 (0.69, 1.48) | 0.99 | 1.31 (0.90, 1.92) | 0.99 |
| Minimum Apnea Duration (sec) | REF | 1.35 (0.59, 3.05) | 0.99 | 2.34 (1.06, 5.16) | 0.56 |
| Mean Heart Rate (BPM) | REF | 1.13 (1.01, 1.25) | 0.39 | 1.14 (1.03, 1.26) | 0.17 |

Note: BPM=beats per minute; pFDR=false discovery rate-corrected p-value; REM=rapid eye movement; SpO_2_=oxygen saturation; SQI=sleep quality index

a. Minimal displacement within ± 7.5 cm; medial displacement ≤ -7.5 cm; lateral displacement ≥ 7.5 cm during preferred walking speed condition

b. Model adjusted for age, sex, race, education, body mass index, and usual gait speed

Supplemental Table 3. Cross-Sectional Associations between Sleep and Step Width Variability Categories, Stratified by Sex (N=72)

|  | **Men (N=35)** | | | **Women (N=37)** | | |
| --- | --- | --- | --- | --- | --- | --- |
|  | **Minimal ^a,b^**  **(RRR, 95% CI)** | **Medial ^a,b^**  **(RRR, 95% CI)** | **Lateral ^a,b^**  **(RRR, 95% CI)** | **Minimal ^a,b^**  **(RRR, 95% CI)** | **Medial ^a,b^**  **(RRR, 95% CI)** | **Lateral ^a,b^**  **(RRR, 95% CI)** |
| Sleep Fragmentation (%) | REF | 0.99 (0.92, 1.07) | 1.06 (0.99, 1.14) | REF | 1.02 (0.95, 1.10) | 1.06 (0.99, 1.13) |
| Stable Sleep (%) | REF | 1.02 (0.94, 1.11) | **0.88 (0.79, 0.98)** | REF | 1.00 (0.93, 1.06) | 0.97 (0.92, 1.03) |
| SQI (%) | REF | 0.99 (0.91, 1.08) | **0.86 (0.76, 0.97)** | REF | 1.01 (0.93, 1.08) | 0.96 (0.89, 1.03) |
| REM Sleep (%) | REF | 0.84 (0.68, 1.02) | **0.80 (0.64, 0.99)** | REF | 0.97 (0.79, 1.20) | 0.93 (0.77, 1.11) |
| Mean SpO_2_ (%) | REF | 1.24 (0.61, 2.51) | 1.21 (0.60, 2.47) | REF | 0.93 (0.57, 1.51) | 1.48 (0.90, 2.45) |
| Minimum Apnea Duration (sec) | REF | 1.41 (0.61, 3.25) | 2.12 (0.90, 4.97) | REF | 0.46 (0.02, 9.49) | 12.55 (0.84, 187.0) |
| Mean Heart Rate (BPM) | REF | 1.09 (0.93, 1.28) | 1.18 (0.99, 1.40) | REF | **1.34 (1.06, 1.70)** | **1.25 (1.01, 1.55)** |

Note: BPM=beats per minute; REM=rapid eye movement; SpO_2_=oxygen saturation; SQI=sleep quality index

a. Minimal displacement within ± 7.5 cm; medial displacement ≤ -7.5 cm; lateral displacement ≥ 7.5 cm during preferred walking speed condition

b. Model adjusted for age, sex, race, education, body mass index, and usual gait speed

Supplemental Table 4. Cross-Sectional Associations between Sleep and Step Width Variability Categories, Stratified by Cognitive Status (N=72)

|  | **CDR=0 (N=56)** | | | **CDR≥0.5 (N=16)** | | |
| --- | --- | --- | --- | --- | --- | --- |
|  | **Minimal ^a,b^**  **(RRR, 95% CI)** | **Medial ^a,b^**  **(RRR, 95% CI)** | **Lateral ^a,b^**  **(RRR, 95% CI)** | **Minimal ^a,b^**  **(RRR, 95% CI)** | **Medial ^a,b^**  **(RRR, 95% CI)** | **Lateral ^a,b^**  **(RRR, 95% CI)** |
| Sleep Fragmentation (%) | REF | 1.00 (0.94, 1.06) | 1.05 (0.997, 1.10) | REF | 1.04 (0.90, 1.19) | 1.15 (0.97, 1.37) |
| Stable Sleep (%) | REF | 1.03 (0.97, 1.09) | 0.96 (0.91, 1.01) | REF | 0.90 (0.73, 1.10) | 0.36 (0.08, 1.71) |
| SQI (%) | REF | 1.00 (0.94, 1.07) | **0.92 (0.86, 0.98)** | REF | 0.93 (0.76, 1.12) | 0.69 (0.43, 1.12) |
| REM Sleep (%) | REF | 0.89 (0.76, 1.05) | 0.89 (0.78, 1.02) | REF | 0.80 (0.48, 1.34) | 0.64 (0.33, 1.21) |
| Mean SpO_2_ (%) | REF | 1.01 (0.65, 1.58) | 1.28 (0.87, 1.89) | REF | 0.72 (0.23, 2.21) | 1.23 (0.42, 3.55) |
| Minimum Apnea Duration (sec) | REF | 0.74 (0.23, 2.42) | 2.68 (0.88, 8.16) | REF | Did not converge | |
| Mean Heart Rate (BPM) | REF | 1.07 (0.96, 1.19) | 1.10 (0.996, 1.21) | REF | 1.28 (0.83, 1.97) | 1.25 (0.83, 1.88) |

Note: BPM=beats per minute; REM=rapid eye movement; SpO_2_=oxygen saturation; SQI=sleep quality index

a. Minimal displacement within ± 7.5 cm; medial displacement ≤ -7.5 cm; lateral displacement ≥ 7.5 cm during preferred walking speed condition

b. Model adjusted for age, sex, race, education, and body mass index

Supplemental Table 5. Cross-Sectional Associations between Sleep and Absolute Value of Step Width Variability (N=72)

|  | **Model 1 ^a^**  **(β, 95% CI)** | **Model 2 ^a^**  **(β, 95% CI)** |
| --- | --- | --- |
| Sleep Fragmentation (%) | 0.12 (-0.06, 0.30) | 0.098 (-0.10, 0.30) |
| Stable Sleep (%) | -0.020 (-0.20, 0.16) | -0.0086 (-0.21, 0.19) |
| SQI (%) | -0.13 (-0.34, 0.08) | -0.11 (-0.33, 0.12) |
| REM Sleep (%) | **-0.74 (-1.20, -0.27)** | **-0.81 (-1.32, -0.30)** |
| Mean SpO_2_ (%) | 0.27 (-1.34, 1.88) | 0.0090 (-1.74, 1.76) |
| Minimum Apnea Duration (sec) | 0.27 (-0.68, 1.21) | 0.067 (-0.96, 1.09) |
| Mean Heart Rate (BPM) | 0.19 (-0.16, 0.53) | 0.32 (-0.06, 0.68) |

Note: BPM=beats per minute; REM=rapid eye movement; SpO_2_=oxygen saturation; SQI=sleep quality index

a. Model 1: unadjusted; Model 2: adjusted for age, sex, race, education, body mass index, and usual gait speed

b. Indicator for sleep apnea hypopnea index (AHI) at 3% during stable non-REM sleep

Supplemental Figure 1. Comparison of SleepImage Reports for Participants with High versus Low Sleep Quality


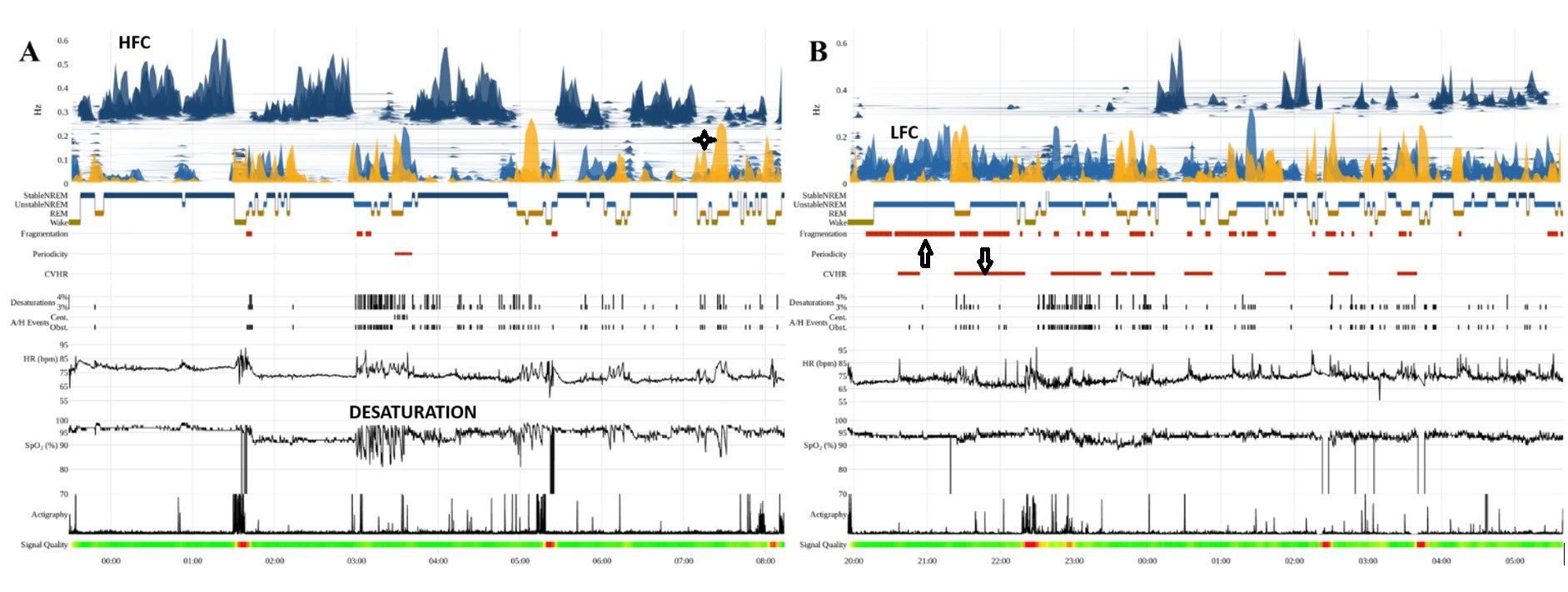


Note: CVHR=cyclic variation in heart rate; HFC=high frequency coupling; LFC=low frequency coupling; PPG=photoplethysmogram; REM=rapid eye movement; SQI=Sleep Quality Index; VLFC=very low frequency coupling

A. PPG Sleep Spectrogram for individual with high SQI (80). High sleep quality is evidenced by large periods of HFC, indicating stable sleep. REM or wake is reflected by areas with VLFC (starred); when activity is low or absent, it is most likely REM sleep, but quiet wake around sleep onset may also mimic REM sleep.

B. PPG Sleep Spectrogram for individual with low SQI (30). Low sleep quality is evidenced by reduced HFC, increased LFC,

increased fragmentation (arrow pointing up), and increased CVHR (arrow pointing down).

Oxygenation (DESATURATION) is abnormal is the person with good sleep quality. Thus, this good or poor sleep quality can be dissociated from sleep apnea. The green bar reflects signal quality, which is excellent in the PPG Sleep Spectrograms for both individuals.
